# Neutrophils Increase or Reduce Parasite Burden in *Trypanosoma cruzi*-Infected Macrophages, Depending on Host Strain: Role of Neutrophil Elastase

**DOI:** 10.1371/journal.pone.0090582

**Published:** 2014-03-05

**Authors:** Tatiana Luna-Gomes, Alessandra A. Filardy, Juliana Dutra B. Rocha, Debora Decote-Ricardo, Isabel Ferreira LaRocque-de-Freitas, Alexandre Morrot, Patrícia T. Bozza, Hugo C. Castro-Faria-Neto, George A. DosReis, Marise P. Nunes, Célio G. Freire-de-Lima

**Affiliations:** 1 Instituto de Biofísica Carlos Chagas Filho, Universidade Federal do Rio de Janeiro, Rio de Janeiro, Brazil; 2 Instituto de Veterinária, Universidade Federal Rural do Rio de Janeiro, Seropédica, Rio de Janeiro, Brazil; 3 Instituto de Microbiologia Paulo de Góes, Universidade Federal do Rio de Janeiro, Rio de Janeiro, Brazil; 4 Instituto Oswaldo Cruz, FIOCRUZ, Rio de Janeiro, Brazil; Pontificia Universidade Catolica do Rio Grande do Sul, Brazil

## Abstract

Neutrophils are involved in the initial steps of most responses to pathogens and are essential components of the innate immune response. Due to the ability to produce and release various soluble mediators, neutrophils may participate in the regulation of the inflammatory response. Little is known about the role of neutrophils during protozoan infections including infection by *Trypanosoma cruzi*. In the present study we investigated the importance of inflammatory neutrophils on macrophage activation and *T. cruzi* replication *in vitro*, in cells obtained from BALB/c mice and C57Bl/6 mice. Co-cultures of BALB/c apoptotic or live neutrophils with infected peritoneal macrophages resulted in increased replication of the parasites and in the production of TGF-β and PGE_2_. The treatment with anti-TGF-β neutralizing antibody and COX inhibitor blocked the parasite replication *in vitro*. On the other hand, co-cultures of *T. cruzi* infected macrophages with live neutrophils isolated from C57BL/6 mice resulted in decreased number of trypomastigotes in culture and increased production of TNF-α and NO. The addition of anti-TNF-α neutralizing antibody or elastase inhibitor resulted in the abolishment of macrophage microbicidal effect and increased parasite replication. Addition of elastase to infected macrophages reduced the replication of the parasites, and on the other hand, addition of a selective inhibitor of iNOS increased parasite growth, suggesting the role of NO in this system. Our findings reveal that neutrophils may regulate *T. cruzi* experimental infection and determine susceptibility and resistance to infection.

## Introduction

Neutrophils are among the first cells to be recruited to the infection site and are important in controlling the host defense through oxidant and protease-dependent mechanisms [1], [2], [3]. These cells also provide an important link between innate and adaptive immunity during parasite infections [4], [5]. Neutrophils can interact with different kinds of cells, like monocytes, dendritic cells, T and B cells through cell-cell contact or secreted products, driving inflammatory responses involved in host defense and tissue repair [5].

Inflammatory neutrophils secrete proteases, chemokines and soluble mediators that regulate inflammation. However, activated neutrophils have a short lifespan and undergo constitutive apoptosis, leading to their phagocytic removal by macrophages [6], [7]. Apoptotic cell clearance interrupts the release of inflammatory factors and administration of apoptotic cells accelerate resolution of inflammation [8]. Cells undergoing apoptosis expose ligands for a set of conserved receptors on macrophage surfaces. These scavenger receptors trigger production of anti-inflammatory mediators such as PGE_2_ and TGF-β and inhibit pro-inflammatory cytokine production [9]. TGF-β down-regulates microbicidal factors, and up-regulates the synthesis of polyamines, leading to an increased replication of intracellular parasites [10]. This mechanism is related to PGE_2_, since cyclooxygenase inhibitors block intracellular parasites growth *in vitro* and drastically reduce TGF-β production [11], [12]. Once the cell becomes apoptotic, it is generally removed *in situ* by macrophages in a quiet, almost invisible fashion, *i.e.* the process does not induce a local tissue reaction. In fact, the recognition and removal of apoptotic cells are normally both anti-inflammatory and anti-immunogenic [10], [13], [14], [15], [16].

Neutrophils have been implicated in different models of intracellular infections including Listeria, Toxoplasma, Mycobacterium and Leishmania [17], [18], [19], [20]. However, the involvement of neutrophils in *T. cruzi* infection has been poorly explored, and little information can be found about this interaction. In the present study we investigated the importance of inflammatory neutrophils on *T. cruzi* replication in BALB/c peritoneal macrophages (used as susceptible model) and C57BL/6 peritoneal macrophages (used as resistant model). We found that, in susceptible BALB/c macrophages, interaction with inflammatory and apoptotic neutrophils increase *T. cruzi* growth through PGE_2_ and TGF-β production. In contrast, inflammatory neutrophils from C57BL/6 mice induced parasite killing. Neutrophil elastase (NE), TNF-α and nitric oxide (NO) are involved in this pathway by inducing microbicidal activity in infected macrophages. These results suggest that decreased inflammatory neutrophils play a role in macrophage regulation of parasite growth.

## Materials and Methods

### Antibodies, cytokines and inhibitors

Neutralizing anti-TGF-β and normal chicken IgY (R&D System), anti-TNF-α, rat IgG1 isotype control (BioSource Europe, Nivelles), were used at 10 µg/mL. Macrophage monolayers were treated with 10 µg/ml aspirin (Sigma-Aldrich), or equivalent dosage of solvent (ethanol). Monolayers were also treated with the specific NE inhibitor methoxysuccinyl-Ala-Ala-Pro-Val-chloromethylketone (MeoSuc-AAPV-CMK; Calbiochen-Novabiochem, La Jolla, CA), control collagenase inhibitor Z-Pro-D-Leu-D-Ala-NHOH (Calbiochem-Novabiochem), both at 10 µg/ml, or equivalent dosage of solvent (DMSO) alone. NO synthase (NOS) inhibitor L-NIL was used at 50 µM/well. Lipopolysaccharide (LPS, *Escherichia coli* 0111:B4) was from List Biological Laboratories, was used at 100 ng/ml.

### Macrophages and *T. cruzi* infection

Primary BALB/c or C57BL/6 peritoneal macrophages (2×10^5^ adherent cells/well in 24-well culture vessels) were infected overnight (ON) with chemically induced metacyclic forms of *T. cruzi* clone Dm28c, obtained as described [21], at a 5∶1 parasite:cell ratio in 1 ml of complete culture medium containing 10% FCS at 37°C. In the following day (day 1), monolayers were extensively washed to remove extracellular parasites and cultured with complete culture medium containing 1% Nutridoma instead of FCS. This study was carried out in strict accordance with the recommendations in the Guide for the Care and Use of Laboratory Animals of the National Institutes of Health (USA). The protocol was approved by the Committee on the Ethics of Animal Experiments of the Health Science Center of the Federal University of Rio de Janeiro (CEUA-CCS, Permit Number: IMPPG 038-05/16) and all efforts were made to minimize suffering.

### Apoptotic neutrophils and co-culture with macrophages

Neutrophils were obtained 7 h after i.p. injection of 1 ml 3% sodium thioglycolate broth (Sigma Chemical Co., MO). Neutrophils elicited by thioglycollate were washed and incubated in DMEM (Sigma Chemical Co., MO) for 1 h at 37°C in humid atmosphere containing 7% CO_2_ in tissue culture flasks (Corning) to remove adherent macrophages. Non-adherent cells (80–90% neutrophils) were either used directly or aged by incubation ON in DMEM without SFB at 37°C in humid atmosphere containing 7% of CO_2_, to induce apoptosis. Aged Gr-1+ cells contained 90% Annexin V+, propidium iodide-negative cells. Apoptotic cells were washed in cold medium before use. Apoptotic or live neutrophils were added at a 10∶1 ratio (1×10^6^), in the presence or absence of Abs, solvents, and reagents. After 7 to 10 days of culture, the number of trypomastigotes released was evaluated using a Neubauer chamber. To evaluate if the effect depends on cell contact, C57BL/6 murine peritoneal macrophages were infected and incubated with live neutrophils separated by membranes (Millicell Culture Plate Inserts 0.22 nm). The number of trypomastigotes released was counted after 7 days of culture in Neubauer chambers. All cultures were done in DMEM (Life Technologies), supplemented with 2 mM glutamine, 5×10^−5^ M 2-ME, 10 µg/ml gentamicin, sodium pyruvate, MEM nonessential amino acids, 10 mM HEPES buffer, and 1% v/v Nutridoma-SP (Boehringer Mannheim).

### Determination of mediators

The concentrations of cytokines in the supernatant obtained from co-cultures of infected macrophages and live or apoptotic neutrophils were quantified after 24 hours of incubation by the method of sandwich immunoassay (ELISA) according to methodology recommended by the manufacturer (R&D). The optical density was obtained by reading in a plate spectrophotometer (VERSAMAX MICROPLATES Reader Molecular Devices, USA). The concentrations of cytokines were calculated from a standard curve of recombinant cytokines. The determination of PGE_2_ (Analysis of absorption by binding enzyme immunoassay) was used EIA kit according to methodology recommended by the manufacturer (Cayman Chemical, Amn Anbor, MZ).

### Evaluation of nitric oxide

Nitric oxide (NO) produced by peritoneal macrophages, co-cultured or not with neutrophils was quantified by the presence of nitrite accumulated in the supernatant of cultures using the Griess colorimetric method described by Kwon et al [22].

### Statistical analysis

Statistical analysis was performed in the program GraphPad InStat version 3.01 (San Diego, USA). Data were analyzed by the method of Student t or ANOVA. Differences with a *p* value 0.05 or lower were considered significant. Data were analyzed by Student' *t* test for independent samples, using SigmaPlot for Windows. Differences with a *p* value <0.05 were considered significant. For parasite loads *in vivo*, counts for left and right draining lymph nodes were first normalized by log transformation, and paired *t* tests were used instead.

## Results

### Interaction with neutrophils regulates replication of *T. cruzi* in macrophages from resistant and susceptible mice

**Figure 1:**
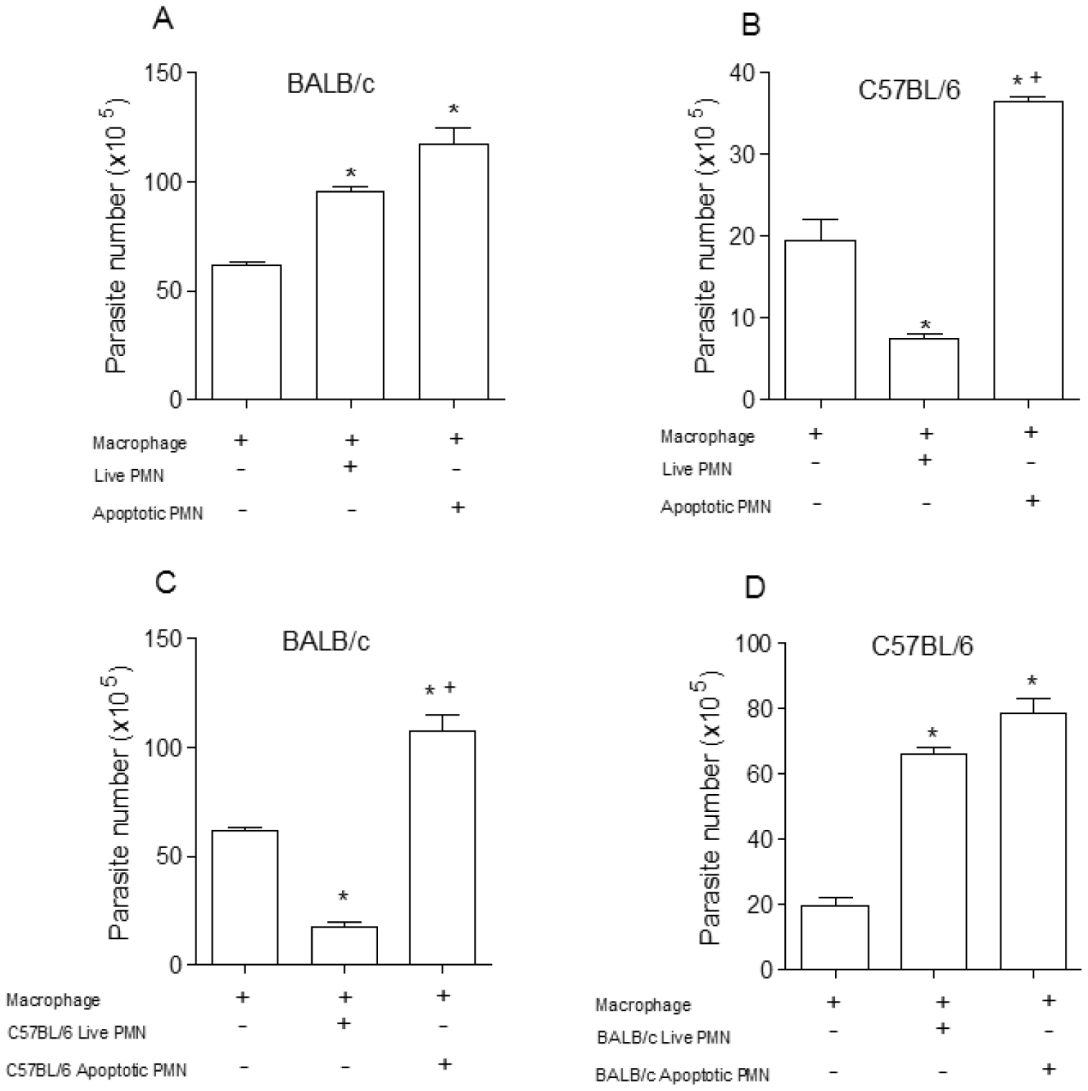
Effect of live or apoptotic neutrophils co-cultured with macrophages infected with *T. cruzi*. Co-culture of macrophages from BALB/c (A) and C57BL/6 (B) mice that were infected *in vitro* with 10^5^ metacyclic trypomastigotes and cultured with syngeneic live or apoptotic neutrophils (PMN). Infected macrophages from BALB/c (C) and C57BL/6 (D) were co-cultured with live or apoptotic neutrophils of different strains. After 7 days the released parasites were counted in a Neubauer chamber. Measurements were performed in triplicate from three different experiments. Statistical significance was determined by ANOVA. When *p≤0.05 compared to infected macrophage and +p≤0.05 compared with alive PMN.

**Figure 2:**
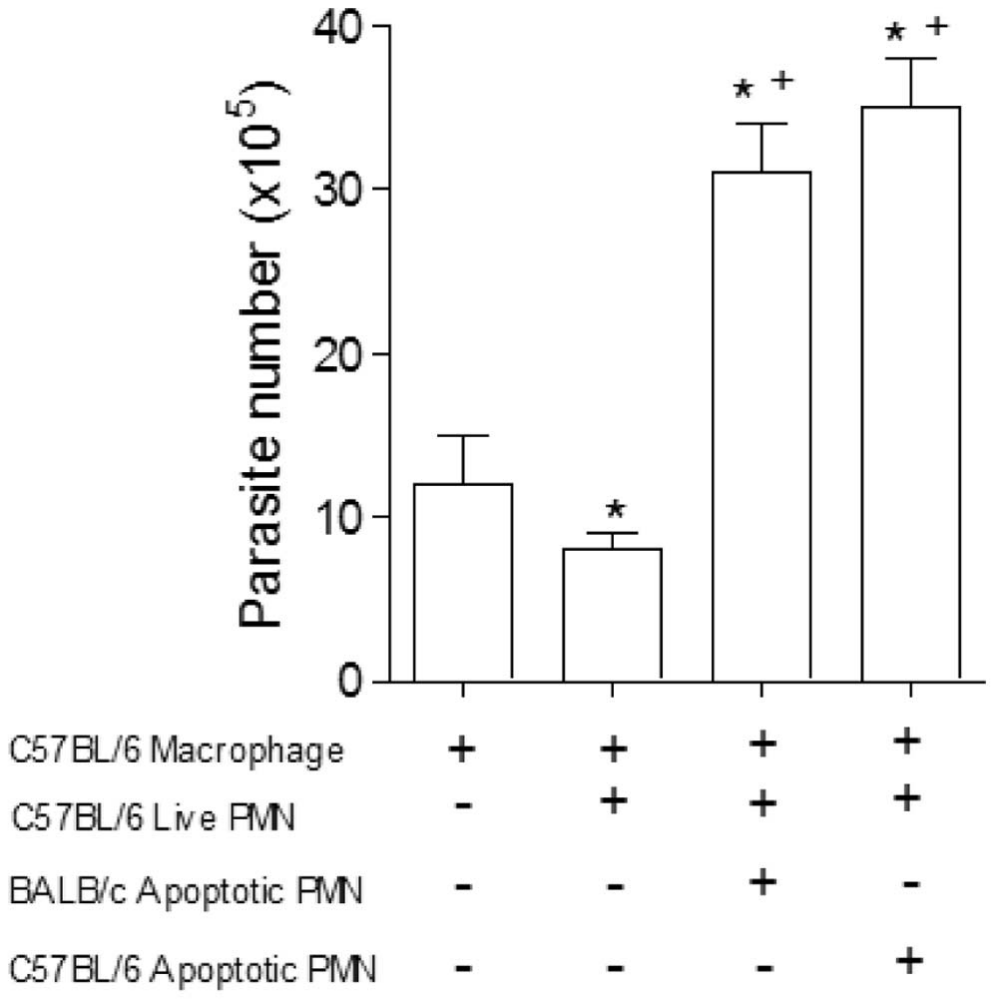
Interference of apoptotic neutrophils on macrophages infected with *T. cruzi* with live C57BL/6 neutrophils. Co-culture of BALB/c peritoneal macrophages with live and apoptotic neutrophils from BALB/c or C57BL/6. After 7 days the parasites released were counted in a Neubauer chamber. Measurements were performed in triplicate from three different experiments. Statistical significance was determined by ANOVA. When *p≤0.05 compared to infected macrophage and +p≤0.05 compared with alive PMN from C57BL/6.

Live or apoptotic inflammatory neutrophils were co-cultured with *T. cruzi* clone Dm28c infected macrophages from BALB/c or C57BL/6 mice, susceptible and resistant model to infection with *T. cruzi* clone Dm28c respectively (data not shown). *In vitro* analysis of the effects of neutrophils on *T. cruzi* infected macrophages revealed that either apoptotic or live neutrophils markedly exacerbated *T. cruzi* replication in BALB/c macrophages (Figure 1a), but inflammatory neutrophils almost eliminated *T. cruzi* when co-cultured with C57BL/6 macrophages, in strain-specific effects (Figure 1b). To determine whether this effect was dependent on specific interaction between macrophages and neutrophils we repeated the same experiment using neutrophils and macrophages from mixed mice strains. BALB/c macrophages co-cultured with viable C57BL/6 neutrophils killed *T. cruzi* with marked efficiency, but the result was totally reversed when apoptotic C57BL/6 neutrophils were added to the cultures (Figure 1c). Differently, both live and apoptotic neutrophils from BALB/c mice exacerbated *T. cruzi* multiplication in C57BL/6 macrophages (Figure 1d). As showed in figure 2, the trypanocidal activities induced by inflammatory neutrophil from C57BL/6 were reversed in the presence of apoptotic neutrophils from BALB/c or C57BL/6. Apoptotic cells are known to induce an anti-inflammatory and anti-immunogenic response, mediated in part by their induction of active TGF-β in responding cells. Apoptotic cells are rapidly engulfed by adjacent tissue cells or macrophages before they can release pro-inflammatory/proimmunogenic intracellular contents. In addition, recognition of the apoptotic cells is actively anti-inflammatory and anti-immunogenic with generation of anti-inflammatory mediators such as TGF-β and anti-inflammatory eicosanoids. The requirement for TGF-β was also shown in the inhibition of thromboxane synthase and thromboxanes, of 5-lipoxygenase and sulfidopeptide leukotrienes, as well as of inducible nitric-oxide synthase and NO [9], [10].

### TGF-β, TNF-α, NO and PGE_2_ controls *T. cruzi* infection *in vitro*

**Figure 3:**
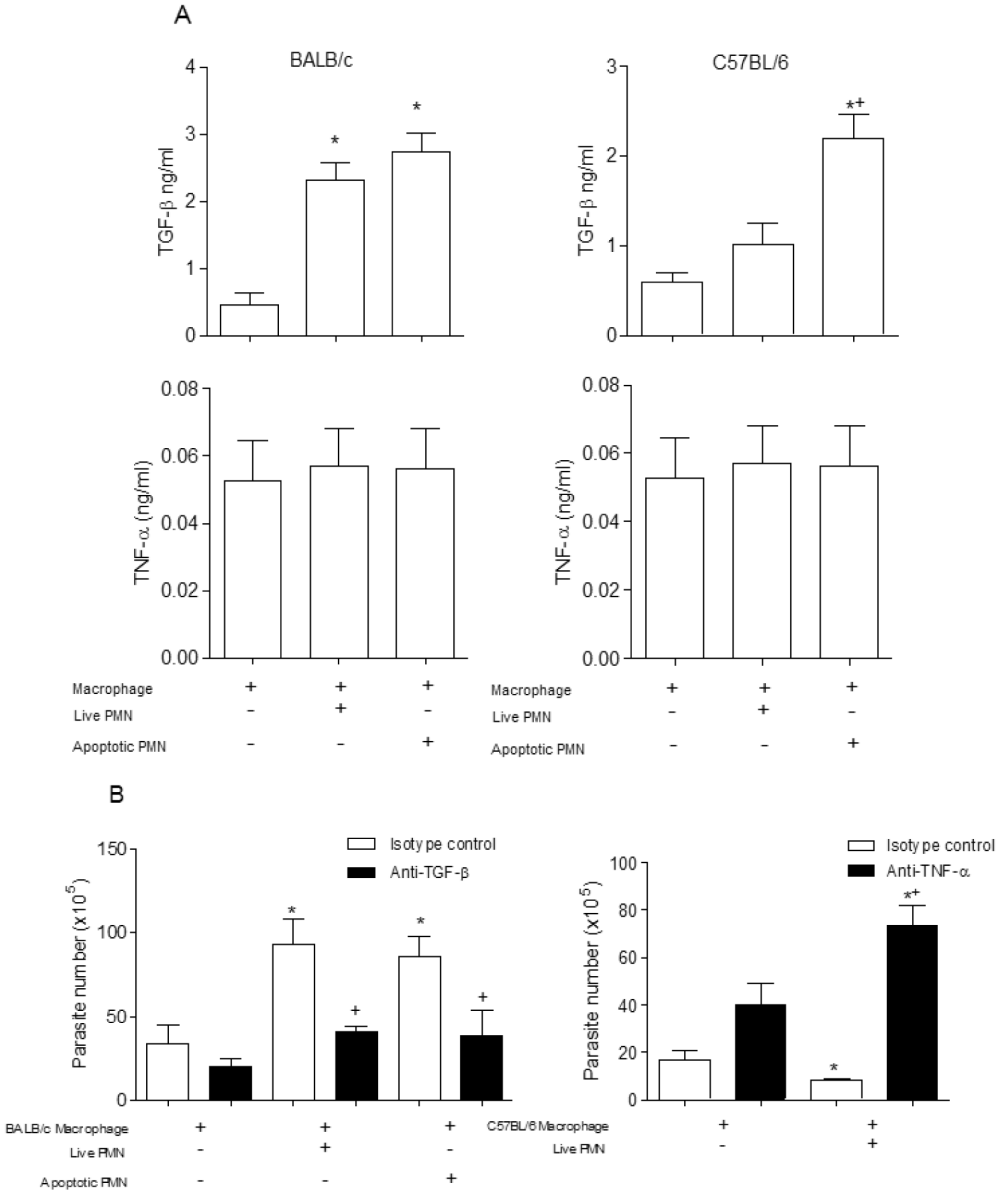
Cytokine production. (A) The levels of cytokines (TGF-β and TNF-α) were determined by enzyme immunoassay in samples of supernatants from co-cultures of macrophages with neutrophils. The tests were performed with samples obtained after 24 hours. Measurements were performed in triplicate of three different experiments. (B) Co-cultures of macrophages infected with live neutrophils and added monoclonal antibodies anti-TGF-β (10 µg/ml) or anti-TNF-α (10 µg/ml). After 7 days the parasites release were counted in a Neubauer chamber. Measurements were performed in triplicate of three different experiments. Statistical significance was determined by ANOVA. When *p≤0.05 compared to infected macrophage and +p≤0.05 compared with isotype control.

Phagocytosis of apoptotic cells induces secretion of regulatory factors [9]. We measured production of TGF-β and TNF-α induced by *T. cruzi* infected macrophages, co-cultured with apoptotic or live neutrophils. Exposure to live or apoptotic BALB/c neutrophils selectively induced TGF-β production by infected macrophages, but not TNF-α. Live C57BL/6 neutrophils induced TNF-α, but not TGF-β. Conversely, apoptotic C57BL/6 neutrophils induced marked TGF-β production by infected macrophages (Figure 3a). To investigate whether TGF-β is required to exacerbated *T. cruzi* growth, we neutralized TGF-β activity. The use of neutralizing anti-TGF-β antibody completely abolished *T. cruzi* replication induced by live or apoptotic BALB/c neutrophils, compared with isotype control (Figure 3b). To investigate the role of TNF-α in *T. cruzi* killing, we neutralized TNF-α activity induced by live C57BL/6 neutrophils. A neutralizing anti-TNF-α antibody completely abolished tripanocidal activity (Figure 3b).

**Figure 4:**
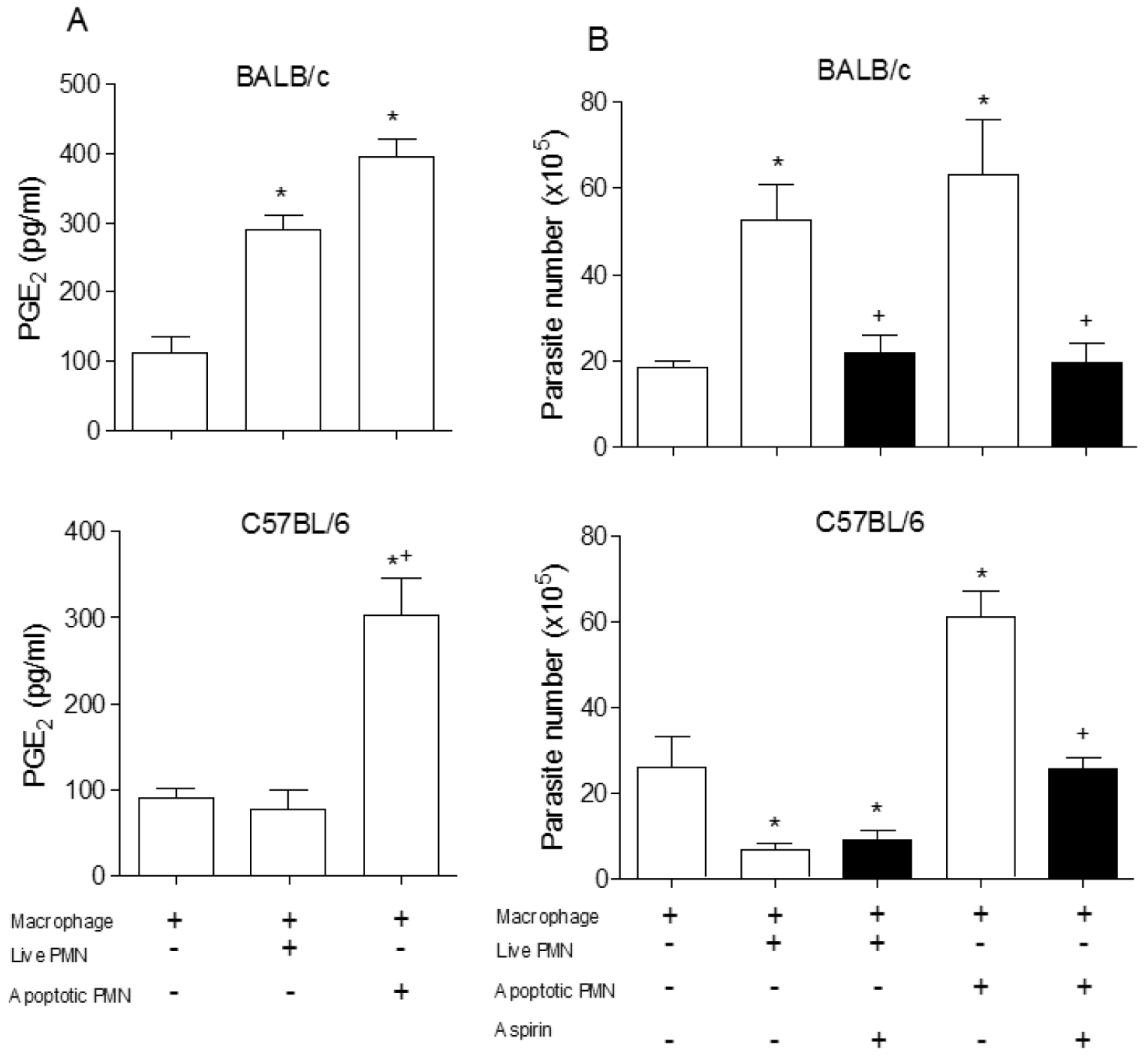
Participation of PGE_2_ in the modulation of infection. (A) PGE_2_ levels were determined in samples of supernatants from co-cultures of BALB/c mice with or without alive and apoptotic neutrophils determined by enzyme immunoassay (EIA). The assays were performed with samples obtained after 24 hours of culture. Measurements were performed in triplicate from three different experiments. (B) Co-cultures of macrophages infected with neutrophils were incubated with aspirin (10 µg/ml) and their solvent (DMSO) and 7 days after the released parasites were counted in Neubauer chamber. Measurements were performed in triplicates of three different experiments. Statistical significance was determined by ANOVA (A) and *t* student test (B). When *p≤0.05 compared to infected macrophage and +p≤0.05 compared to the apoptotic PMN.

Growth of *T. cruzi* driven by uptake of apoptotic T cells depends on TGF-β and PGE_2_ production [10]. In addition, PGE_2_ is required for TGF-β production induced by uptake of apoptotic cells [10], [11]. We tested the role of PGE_2_ in *T. cruzi* replication induced by inflammatory or apoptotic neutrophils. We observed a higher production of PGE_2_ in the presence of BALB/c live or apoptotic neutrophils, in contrast to cultures with live C57BL/6 neutrophils (Figure 4a). In addition, cyclooxygenase inhibitor aspirin blocked the release of *T. cruzi* induced by live BALB/c neutrophils and apoptotic neutrophils from both mouse strains (Figure 4b).

**Figure 5:**
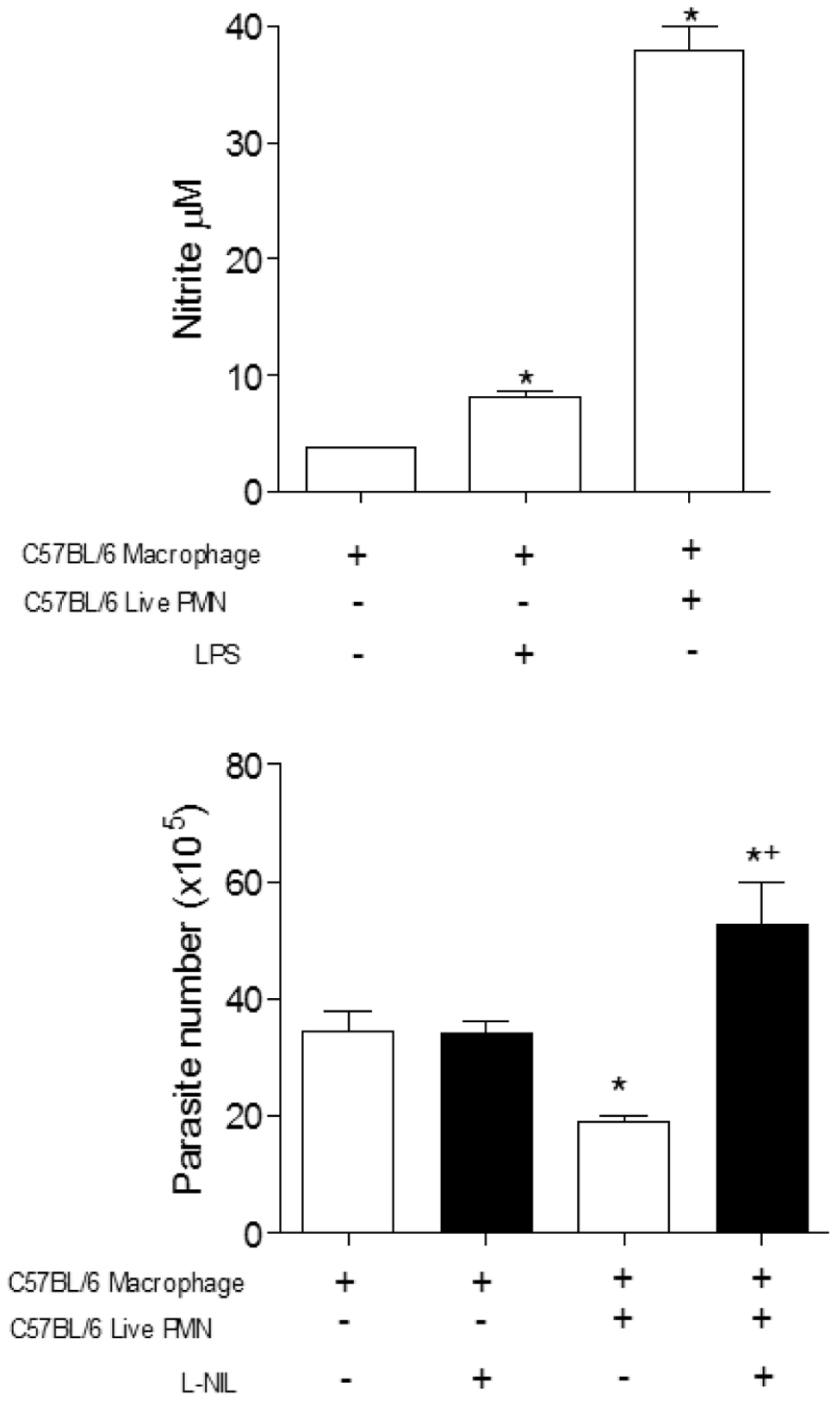
The microbicidal effect of neutrophils from C57BL/6 is dependent on Nitric Oxide. (A) Increased production of nitrite found in the supernatant co-cultures of neutrophils from C57BL/6 after 24 hours. (B) Inhibition of NO production in the presence of selective inhibitor (L-NIL) and interference on the parasite growth in macrophages from C57BL/6. After 7 days the released parasites were counted in a Neubauer chamber. Measurements were performed in triplicate from three different experiments. Statistical significance was determined by ANOVA. When *p≤0.05 compared to infected macrophage and +p≤0.05 compared with alive PMN.

We next investigated whether NO was involved in parasite killing. Our results revealed a greater production of nitrite when infected macrophages were co-cultured with live neutrophils from C57BL/6 (Figure 5a). In agreement, the specific iNOS inhibitor L-NIL increased *T. cruzi* release in macrophages co-cultured with C57BL/6 neutrophils (Fig. 5b).

### Trypanocidal Activity induced by NE

**Figure 6:**
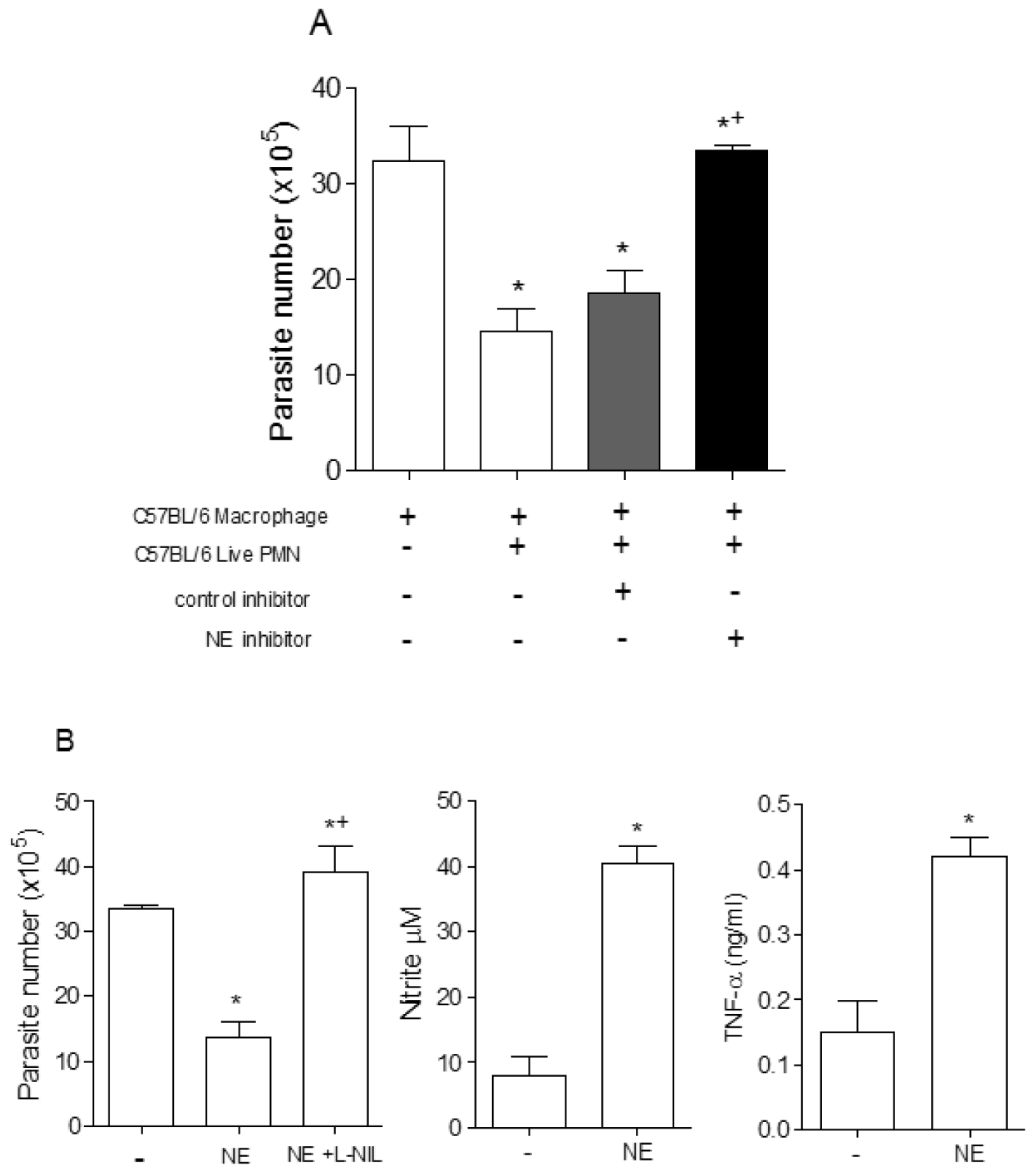
Neutrophil elastase potentiates the microbicidal response of *T. cruzi in vitro*. (A) Parasites released in infected C57BL/6 macrophages cultures alone or in presence of solvent (DMSO), control inhibitor (collagenase inhibitor Z-Pro-D-Leu-AAPV-D-Ala-NHOH; used at 10 µg/ml) or NE inhibitor (MeOSuc-AAPV-cmk; used at 10 µg/ml). (B) Neutrophil elastase (100 ng/ml) was added to infected cultured macrophages in the presence or absence of selective iNOS inhibitor (L-NIL). The number of trypomastigotes was counted in a Neubauer chamber after 7 days of culture. Production of TNF-α and NO released into the supernatant by *T. cruzi* infected macrophages after 24 h incubation in medium alone or in the presence of neutrophil elastase, at 100 ng/ml. Measurements were performed in triplicates of three different experiments. Statistical significance was determined by ANOVA (A) or t test (B).

Serine protease NE is a known inducer of TNF-α production by murine and human macrophages [23]. In our study we used inflammatory neutrophils that are normally degranulating cells. Then, we decided to investigate the involvement of serine proteases released by inflammatory C57BL/6 neutrophils in parasite killing. Using a transwell system, we found that the trypanocidal activity observed in macrophages co-cultured with inflammatory C57BL/6 neutrophils was independent on cell contact (Figure S1). Furthermore, the addition of the specific NE inhibitor, MeOsuc-AAPV-CMK, abolished the killing induced by inflammatory C57BL/6 neutrophils (Figure 6a). Given these results, we investigated the effects of purified NE on induction of a microbicidal state in peritoneal macrophages. Initially, we found that addition of NE at dose of 1 µg/mL induced morphological changes in infected macrophages (data not shown). However, at doses of 100–500 ng/mL of NE, we observed dramatic membrane spreading in macrophages without any sign of cell death (data not shown). Furthermore, addition of purified NE reduced *T. cruzi* trypomastigote release by infected macrophages. In agreement, addition of purified NE induced NO and TNF-α production by infected macrophages (Figure 6b).

## Discussion

Neutrophils are the first cells to migrate to inflammatory sites and thus may regulate the immune response against infectious agents. These cells have short half-life and are constitutively programmed to die by apoptosis [7]. For a long time the role of neutrophils in immunoregulation received little attention. However, evidences from different groups [24], [25], [26], [27] suggests that neutrophils can contribute significantly to the immune response by modulating both cellular and humoral immunity. Neutrophils are also implicated as an important source of cytokines [28], [29] establishing a link between innate and adaptive immunity response [4], [5]. These cells can also facilitate the invasion of macrophages by *L. major in vitro* and *in vivo* [30], [31].

Neutrophils play either protective or deleterious roles during Chagas' disease according to the mouse strain studied [25]. However, this conclusion was based on depletion of Gr-1+ cells that has been shown to comprise not only neutrophils but also dendritic cells, monocytes, macrophages and lymphocytes and therefore, the effects described can not be related only to neutrophils [32]. Consequently, the specific role of neutrophils during *T. cruzi* infection remained elusive. In this study we evaluated the role of live or apoptotic neutrophils cultured with *T. cruzi* infected macrophages. Our results demonstrated that apoptotic neutrophils increased parasite replication in macrophages regardless of the macrophage lineage employed. Phagocytosis of apoptotic cells by macrophages can suppress inflammation, favoring intracellular replication of parasites through the release of antiinflammatory mediators [10]. However, little is known about the role of neutrophils in experimental Chagas' disease. Indeed, interaction of neutrophils with macrophages plays an important role in regulating the host response to infection by another intracellular parasite, *L. major* [19]. We also demonstrated that the increase in parasite replication within macrophages was related to the production of TGF-β and PGE_2_ [10], [33], suggesting that the addition of the neutrophils is exerting an immunosuppressive effect *in vitro*. Phagocytosis of apoptotic neutrophils inactivates macrophages through the secretion of prostaglandin and TGF-β [11], [34], [12]. In addition, TGF-β is an antiinflammatory mediator and participates in the metabolism of L-arginine, favoring the production of arginase, which is involved in the activity of ornithine decarboxylase [10]. The intracellular stage of *T. cruzi* requires this host cell enzyme and the synthesis of polyamines to multiply inside the cell [10]. It is possible that this mechanism is involved in the exacerbation of parasite growth observed in the present study. In agreement, both anti TGF-β and aspirin inhibited parasite replication.

Interestingly, the same phenomenon observed for apoptotic neutrophils was also observed when live neutrophils of BALB/c were added to the cultures. Co-culture of infected macrophages with BALB/c live neutrophils increased parasite replication *in vitro*. This phenomenon was also mediated by the production of PGE_2_ and TGF-β and could be reversed in the presence of anti-TGF-β and aspirin. The immunosuppressive role of neutrophils during an infection has already been described [19] and the role of BALB/c anti-inflammatory neutrophils could be another factor involved in this strain susceptibility to infection by *T. cruzi*. Our data suggests that neutrophils from BALB/c mice could produce cytokines and chemokines that may play a role in promoting the production of immunoregulatory cytokines by other cells such as macrophages. Our results showed an opposite effect when live neutrophils from C57BL/6 mice were used. Addition of these neutrophils could control intracellular parasite multiplication. This trypanocidal activity was independent on cell contact, and was dependent on the production of TNF-α, NO and elastase. Macrophages which have been co-cultured with C57BL/6 neutrophils showed increased microbicidal activity as observed by increased production of NO, a major factor for the control of intracellular infections [35], [36], [37]. Neutrophils from B6 mice released 2–3 times more NE protein and enzymatic activity into the supernatant, compared with neutrophils from BALB/c mice [38]. Our data demonstrated an inflammatory environment mediated by TNF-α in the co-cultures of live neutrophils from C57BL/6 mice. Furthermore, the significant inhibition of parasite killing by anti-TNF-α indicated the involvement of this cytokine [27]. A modulatory role of elastase was described by Fadok and Chimini [39], who showed the production of pro-inflammatory mediators by macrophages stimulated with elastase. In addition, elastase induces production of TNF-α by macrophages in an NFκB-dependent mechanism [40]. We demonstrated that neutrophil elastase appears to be involved in increased trypanocidal activity. These data were confirmed when elastase was inhibited, and the microbicidal effect induced by live neutrophils from C57BL/6 mice was blocked. Our results indicate that treatment with elastase induces the production of nitric oxide and TNF-α by macrophages, thereby increasing the trypanocidal activity. Further experiments are necessary to understand whether there is a difference in expression and function of neutrophil elastase from C57BL/6 and BALB/c neutrophils. In conclusion, neutrophils derived from BALB/c (live or apoptotic) lead to production of inflammatory mediators such as TGF-β and PGE_2_, which favor the growth of the parasite. On the other hand, live C57BL/6 neutrophils are associated with increased production of neutrophil elastase, thus providing greater activation to macrophages that can better respond to infection with increased production of TNF-α and nitric oxide. Taken together, our results reinforce the importance of neutrophils and their products in modulation of *T. cruzi* infection. In addition, ours results suggests the possible involvement of neutrophils in susceptibility to infection.

## Supporting Information

Figure S1 **The microbicidal effect of co-culture of macrophages infected with** ***T. cruzi*** **and live neutrophils is independent of contact.** Resident macrophages (C57BL/6) were plated in 48-well vessels at 1.5×10^5^ cells/well, in complete culture medium, and infected with ratio of 1∶3 (macrophages:*T. cruzi*). Live neutrophils of C57BL/6 mice (1×10^6^) were either added in the same compartment, or separated by a cell-impermeable culture insert. The number of trypomastigotes was counted in a Neubauer chamber after 7 days of culture. Measurements were performed in triplicates of three different experiments. Statistical significance was determined by *t* test (with p≤0.05).
(TIF)
