# Supplementary figures and images for: Neutrophils Increase or Reduce Parasite Burden in Trypanosoma cruzi-Infected Macrophages, Depending on Host Strain: Role of Neutrophil Elastase
Source: PLoS One. 2014 Mar 5;9(3):e90582. doi: 10.1371/journal.pone.0090582 (PMC3944110; doi:10.1371/journal.pone.0090582)

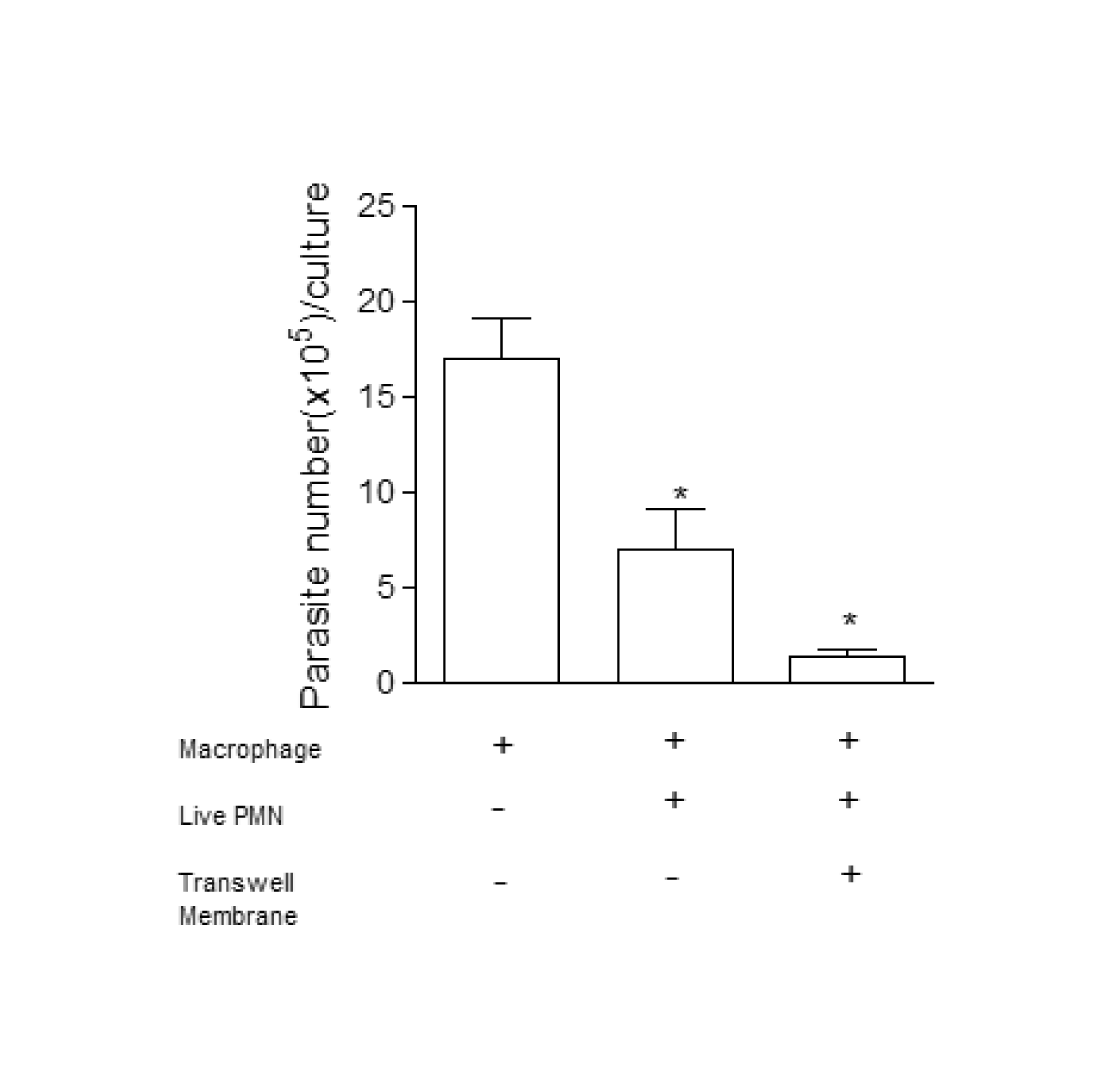

Supplement: Figure S1 — The microbicidal effect of co-culture of macrophages infected with T. cruzi and live neutrophils is independent of contact. Resident macrophages (C57BL/6) were plated in 48-well vessels at 1.5×10^5^ cells/well, in complete culture medium, and infected with ratio of 1∶3 (macrophages:T. cruzi). Live neutrophils of C57BL/6 mice (1×10^6^) were either added in the same compartment, or separated by a cell-impermeable culture insert. The number of trypomastigotes was counted in a Neubauer chamber after 7 days of culture. Measurements were performed in triplicates of three different experiments. Statistical significance was determined by t test (with p≤0.05). (TIF) [file pone.0090582.s001.tif]
